# FAMILY SUPPORT, MOTIVATION, AND PATIENT ADHERENCE TO TUBERCULOSIS TREATMENT: INSIGHTS FROM INDONESIA

**DOI:** 10.21010/Ajidv19i2.5

**Published:** 2025-04-07

**Authors:** NINDREA Ricvan Dana, MING Long Chiau, AGUSTIAN Dede Rahman

**Affiliations:** 1 Department of Medicine, Faculty of Medicine, Universitas Negeri Padang, Bukittinggi, Indonesia, 26181; 2 School of Medical and Life Sciences, Sunway University, Malaysia, 47500; 3 Graduate Institute of Biomedical Sciences, China Medical University, Taichung, Taiwan, 406040

**Keywords:** Family support, Motivation, Treatment adherence, Tuberculosis

## Abstract

**Background:**

Tuberculosis (TB) remains a significant public health issue globally, with patient adherence to treatment being critical for successful outcomes.

This study aimed to investigate the roles of family support and motivation in influencing adherence to TB treatment in Indonesia.

**Materials and Methods:**

A cross-sectional study was conducted among patients at four health centers in Padang Municipality, Indonesia, between January and November 2024. A total of 125 respondents diagnosed with drug-sensitive TB and in the second stage of treatment participated. Data were collected through structured interviews using validated questionnaires, assessing family support, motivation, and treatment adherence. Structural Equation Modeling (SEM) was used to analyze the relationships between variables.

**Results:**

The findings revealed significant relationships between family support and motivation (β = 0.931, t-value = 47.016, P<0.001), family support and adherence to TB treatment (β = 0.229, t-value = 2.743, P=0.006), and motivation and adherence to TB treatment (β = 0.775, t-value = 9.334, P<0.001).

**Conclusion:**

Family support and motivation play crucial roles in enhancing patient adherence to TB treatment. Strategies to strengthen family involvement and patient motivation should be integrated into TB control programs to improve treatment outcomes. Further research is recommended to explore additional factors influencing adherence in different contexts.

## Introduction

Tuberculosis (TB) continues to be a critical global health issue, especially in low- and middle-income nations (Nindrea *et al.*, 2024). The World Health Organization (WHO) reports that TB is among the leading causes of mortality worldwide, with approximately 10.6 million new infections and 1.3 million deaths recorded in 2022 (Lestari *et al.*, 2023). Indonesia is among the top three countries contributing to the global TB burden, accounting for approximately 10% of the global TB cases (Lestari *et al.*, 2023). Despite efforts to reduce the TB burden, challenges such as poor treatment adherence, stigma, and limited access to healthcare services persist (Bedingfield *et al.*, 2023; Chen *et al.*, 2020).

Drug-sensitive tuberculosis (DS-TB), the most common form of TB, refers to cases caused by Mycobacterium tuberculosis that respond to first-line anti-TB drugs such as isoniazid and rifampin. DS-TB cases have a generally favorable prognosis when the full 6–8 month treatment regimen recommended by the WHO is completed (Bedingfield *et al.*, 2023; Chen *et al.*, 2020). However, poor adherence to treatment remains a critical challenge, leading to treatment failure, relapse, and, in severe cases, the emergence of multidrug-resistant TB (MDR-TB) (Chowdhury *et al.*, 2023). Ensuring adherence in DS-TB cases is pivotal not only for individual recovery but also for breaking the cycle of TB transmission and preventing the development of drug-resistant strains. In Indonesia, where TB incidence remains high, addressing barriers to adherence in DS-TB is an urgent public health priority (Sazali *et al.*, 2023).

Adherence to TB treatment is critical for successful outcomes. The WHO recommends a 6–8 month treatment regimen for drug-sensitive TB, but many patients fail to complete this due to personal, social, and economic barriers (Sazali *et al.*, 2023; Chowdhury *et al.*, 2023). Research indicates that family support and motivation are significant determinants of TB treatment adherence (Gebremariam *et al.*, 2021; Lutfian *et al.*, 2025). Family support, both emotional and practical, provides TB patients with encouragement, reminders, and assistance throughout the prolonged treatment journey (Dilas *et al.*, 2023; Lutfian *et al.*, 2025). It helps patients overcome barriers such as fatigue, stigma, and financial burdens (Nagarajan *et al.*, 2022; Wong *et al.*, 2021). Similarly, patient motivation shaped by personal awareness of the disease, family encouragement, and healthcare provider support plays a vital role in sustaining adherence to treatment regimens (Yadav *et al.*, 2024; Dilas *et al.*, 2023).

In Indonesia, socio-cultural factors further complicate TB treatment adherence. The strong family-oriented culture can serve as a vital support system for TB patients (Lolong *et al.*, 2023). However, stigma and misconceptions surrounding TB remain prevalent, causing some patients to delay treatment or conceal their condition (Ashaba *et al.*, 2021). Understanding how family support and motivation influence TB treatment adherence in this setting is essential to designing effective interventions (Saidi *et al.*, 2023).

This study aims to investigate the relationship between family support, motivation, and patient adherence to TB treatment in Indonesia. By identifying these key factors, the findings can inform targeted interventions to improve TB treatment outcomes and strengthen the country’s efforts to meet global TB elimination targets by 2030, as outlined in the WHO End TB Strategy.

## Materials and Methods

### Study design and research sample

A cross-sectional study was conducted to examine patients seeking care at four health centers in Padang Municipality, Indonesia. Data collection took place from January to November 2024. The survey focused on patients diagnosed with drug-sensitive TB who had advanced to stage 2, representing individuals with active disease following infection. However, patients in the second stage who were merely exposed without disease progression, along with those diagnosed with extensively drug-resistant tuberculosis (XDR TB) or MDR TB, were excluded from participation. For the sample size calculation, we hypothesized that 91% of patients with family support adhere to tuberculosis treatment (p = 0.91 p=0.91) (Nindrea *et al.*, 2024). With a 95% confidence level and a 5% margin of error, the required sample size was determined to be 125 respondents. Sample selection utilized multistage stratified clustered sampling.

### Variables and operational definitions

The constructs in this study were defined and measured using reliable and validated scales derived from established peer-reviewed journals. Patient adherence to TB treatment was assessed through four questions: whether healthcare providers give clear and timely advice about treatment during visits to the health center, whether patients are aware of their sputum test schedule, whether doctors express concern about disease progression during consultations, and whether patients are asked to provide sputum samples during health center visits (Dilas *et al.*, 2023). Family support was evaluated using five statements: the family provides emotional support during TB treatment, encourages the completion of treatment, shares information to help understand the importance of TB treatment, reminds the patient about their medication schedule, and assists with daily necessities during treatment (Nindrea *et al.*, 2024). Motivation was measured through five statements: understanding the importance of completing TB treatment for recovery, believing that completing treatment improves quality of life, feeling motivated by family support to stay committed to treatment, receiving encouragement from healthcare providers to complete treatment, and not experiencing community stigma that diminishes motivation to complete treatment (Dilas *et al.*, 2023; Nindrea *et al.*, 2024). All variables were measured using a Likert scale. The instruments used in this study to assess patient adherence to TB treatment, family support, and motivation demonstrated robust content and construct validity, with reliability confirmed by a Cronbach’s alpha greater than 0.7, indicating high internal consistency (Dilas *et al.*, 2023; Nindrea *et al.*, 2024).

### Data collection

The study employed a research questionnaire to collect data. Informed consent was obtained from all respondents, who were assured that their participation was entirely voluntary and that they could withdraw at any time without any consequences. To ensure confidentiality, respondents’ identities were anonymized, and no names were disclosed to prevent any potential repercussions.

### Ethical approval

This study received ethical approval from the Research Ethics Committee of General Hospital of Dr. M. Djamil in Padang Municipality, Indonesia (No. DP.04.03/D.XVI.XI/539/2023).

### Data analysis

In this study, descriptive statistics, such as frequencies, percentages, and median values, were utilized. The reliability and validity of the measurement scales were assessed using Cronbach’s alpha, CR, and AVE. Furthermore, SEM was employed to test the research hypotheses, with a significance level set at P < 0.05.

## Results

Table 1 shows that the average age of the respondents was 41.52±3.61 years. Most respondents were male, accounting for 56.0% of the sample. Over half of the participants reported a family income below 2,810,000 IDR (68.8%). The average number of family members was 4.51±0.23. Junior high school was the most frequently reported level of education, and the most common occupation was entrepreneurship, representing 28.0% of the group (Table 1).

**Table 1:** Characteristics of respondents

Variables	Value (n=125)
**Age (years), (mean±SD)**	41.52±3.61
**Sex, f(%)**	
Male	70 (56.0)
Female	55 (44.0)
**Family income based on regional minimum wage, f(%)**	
< 2,810,000 IDR	86 (68.8)
≥ 2,810,000 IDR	39 (31.2)
Number of family members, (mean±SD)	4.51±0.23
Educational level, f(%)	
No schooling	11 (8.8)
Elementary school	27 (21.6)
Junior high school	51 (40.8)
Senior high school	32 (25.6)
University	4 (3.2)
**Occupation, f(%)**	
Government employee	22 (17.6)
Private sector employee	20 (16.0)
Entrepreneur	35 (28.0)
Small-scale merchant	28 (22.4)
Farmer	20 (16.0)

Table 2 demonstrated that all standardized factor loadings were above 0.60 and statistically significant (P<0.05). The reliability of the variables was evaluated through Cronbach’s alpha, with all values exceeding 0.70, confirming the reliability of the variables. Convergent validity of the scales was assessed based on three criteria: first, all indicator loadings were greater than 0.70; second, CR values exceeded 0.8; and third, the AVE for each construct was 0.5 or higher. These findings indicated that the research constructs achieved satisfactory convergent validity (Table 2).

**Table 2:** Findings on confirmatory factor analysis

Constructs/ factors	Standardized factor loadings
*Patient adherence to TB treatment (α=0.926; CR=0.948; AVE=0.819)*	
PAT1 - When visiting the health center, do the health personnel provide you with clear and timely advice about your treatment?	0.914
PAT2 - Are you aware of when your sputum test is scheduled?	0.902
PAT3- Does the doctor express concern about the progression of your disease during consultations?	0.914
PAT4-Are you asked to provide a sputum sample when you visit the health center?	0.890
*Family support (α=0.924; CR=0.944; AVE=0.771)*	
FS1- I feel that my family provides emotional support during my TB treatment	0.932
FS2 - My family encourages me to complete my TB treatment	0.840
FS3 - My family provides information that helps me understand the importance of TB treatment	0.927
FS4 - My family members remind me about my medication schedule	0.735
FS5 - My family helps me with daily necessities while I undergo TB treatment	0.940
*Motivation* *(α=0.938; CR=0.953; AVE=0.804)*	
M1 - I understand the importance of completing TB treatment for my recovery	0.862
M2 - I believe that completing TB treatment will improve my quality of life	0.894
M3 - Support from my family motivates me to stay committed to the treatment	0.916
M4 - Healthcare providers offer encouragement that inspires me to complete the treatment	0.894
M5 - I do not experience stigma from the community that could diminish my motivation to complete the treatment	0.916

α= Cronbach’s alpha

Table 3 evaluated discriminant validity, which reflects the degree to which the three variables are empirically distinct from one another. To establish discriminant validity, the AVE of each latent construct needed to exceed the squared correlations between that construct and all other latent constructs. The results confirmed that these criteria for discriminant validity were met (Table 3).

**Table 3:** Findings on discriminant validity

No	Constructs	1	2	3
1	Family support	*0.878*		
2	Motivation	0.490	*0.897*	
3	Patient adherence to TB treatment	0.423	0.563	*0.905*

**Figure 1:**
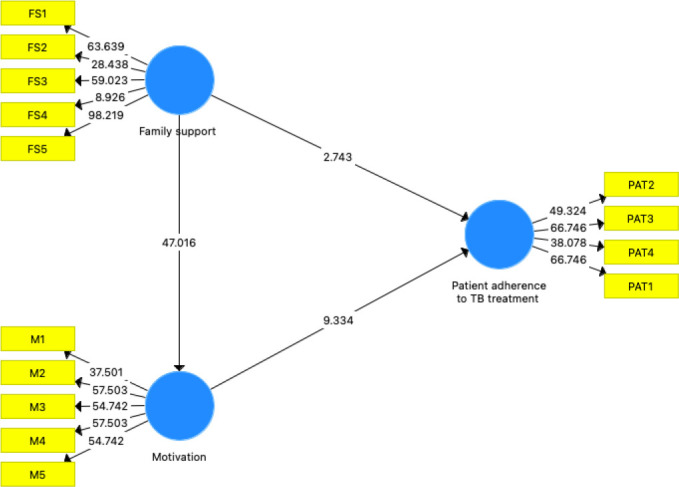
A path analysis of the relationship between family support, motivation, and patient adherence to TB treatment in Indonesia.

Table 4 and Figure 1 depict the relationships between the study variables. A strong association was observed between family support and motivation (β = 0.931, t-value = 47.016, P < 0.001). Additionally, family support was positively linked to patient adherence to TB treatment (β = 0.229, t-value = 2.743, P = 0.006), while motivation also showed a significant positive relationship with patient adherence (β = 0.775, t-value = 9.334, P < 0.001). (Table 4 and Figure 1).

**Tabel 4:** The relationship between family support, motivation, and patient adherence to TB treatment in Indonesia

Path specified	Coefficient (β)	t-value	P-value	Result
Family support -> Motivation	0.931	47.016	<0.001	Accepted
Family support -> Patient adherence to TB treatment	0.229	2.743	0.006	Accepted
Motivation -> Patient adherence to TB treatment	0.775	9.334	<0.001	Accepted

## Discussion

This study highlights the significant roles of family support and motivation in improving adherence to TB treatment. Consistent with previous research, the findings affirm that family support serves as a critical determinant in encouraging patients to adhere to TB treatment regimens (Lutfian *et al.*, 2025). Other studies have similarly demonstrated that emotional, informational, and practical support from family members can significantly influence patient behavior, particularly in chronic disease management (Strom *et al.*, 2012; Hendriyani *et al.*, 2020; Bradshaw *et al.*, 2022). Likewise, motivation, driven by patient awareness and external encouragement, aligns with prior studies showing its pivotal role in fostering adherence to treatment protocols (Nindrea *et al.*, 2020; Fahmi *et al.*, 2021).

The study phenomena underscore the influence of cultural and demographic characteristics on treatment adherence (Widayanti *et al.*, 2023). In Indonesia, family units often play a central role in an individual’s decision-making and daily activities due to collectivist cultural norms (Soemantri *et al.*, 2021). This cultural context likely amplifies the impact of family support on patient motivation and adherence (Febriyanti *et al.*, 2024). The demographic characteristics of the respondents, such as their predominantly low-income status and varying educational levels, further emphasize the importance of family support in bridging gaps in healthcare literacy and accessibility (Coombs *et al.*, 2022; Nindrea *et al.*, 2022). Families not only provide emotional support but also help manage logistical challenges, such as reminding patients of appointments and ensuring medication adherence, which are particularly crucial in resource-limited settings (Kvarnström *et al.*, 2021; Cross *et al.*, 2020).

The implications of these findings are far-reaching. Health programs should integrate family-centered approaches into TB care, providing education and resources to families to enhance their supportive roles (Myburgh et al., 2023). Additionally, motivational strategies tailored to individual patient needs, such as counseling or community support, can further strengthen adherence outcomes (Fernandes *et al.*, 2023; Dilas *et al.*, 2023; Nindrea *et al.*, 2019). Public health initiatives should also consider cultural dynamics and address demographic disparities to ensure equitable access and adherence to TB treatment.

The study’s strengths include its robust methodological design, validated measurement tools, and the use of SEM to analyze complex relationships among variables. However, some limitations should be acknowledged. The study was conducted in a single municipality, potentially limiting the generalizability of the findings. Furthermore, the cross-sectional design precludes causal inferences, and the reliance on self-reported data may introduce bias.

Future research should explore longitudinal designs to confirm causal relationships and extend the scope to diverse populations and settings. Additional studies should also investigate how cultural norms and socioeconomic factors intersect with family support and motivation to influence TB treatment adherence. Despite its limitations, this study provides valuable insights into the psychosocial and cultural factors influencing TB treatment adherence, specifically through the lenses of family support and patient motivation. The cultural context, particularly Indonesia’s collectivist norms, amplifies the role of family as a primary source of emotional, informational, and logistical support, directly impacting patients’ adherence behavior. By focusing on these variables, the study emphasizes the importance of tailoring interventions to cultural and familial dynamics to enhance treatment outcomes.

## Conclusion

This study highlights the critical roles of family support and motivation in improving adherence to TB treatment. Family support enhances motivation and adherence through emotional, informational, and practical assistance, particularly in a collectivist cultural context like Indonesia. The findings emphasize the need for family-centered approaches and motivational strategies in TB care, addressing psychosocial and socioeconomic factors to optimize outcomes. Future research should explore these dynamics further across diverse populations and settings to inform more effective TB treatment strategies.
